# Differences in Field Effectiveness and Adoption between a Novel Automated Chlorination System and Household Manual Chlorination of Drinking Water in Dhaka, Bangladesh: A Randomized Controlled Trial

**DOI:** 10.1371/journal.pone.0118397

**Published:** 2015-03-03

**Authors:** Amy J. Pickering, Yoshika Crider, Nuhu Amin, Valerie Bauza, Leanne Unicomb, Jennifer Davis, Stephen P. Luby

**Affiliations:** 1 Civil and Environmental Engineering, Stanford University, Stanford, CA, United States of America; 2 Woods Institute for the Environment, Stanford University, Stanford, CA, United States of America; 3 International Center for Diarrheal Diseases Research, Dhaka, Bangladesh; Institute for Health &amp; the Environment, UNITED STATES

## Abstract

The number of people served by networked systems that supply intermittent and contaminated drinking water is increasing. In these settings, centralized water treatment is ineffective, while household-level water treatment technologies have not been brought to scale. This study compares a novel low-cost technology designed to passively (automatically) dispense chlorine at shared handpumps with a household-level intervention providing water disinfection tablets (Aquatab), safe water storage containers, and behavior promotion. Twenty compounds were enrolled in Dhaka, Bangladesh, and randomly assigned to one of three groups: passive chlorinator, Aquatabs, or control. Over a 10-month intervention period, the mean percentage of households whose stored drinking water had detectable total chlorine was 75% in compounds with access to the passive chlorinator, 72% in compounds receiving Aquatabs, and 6% in control compounds. Both interventions also significantly improved microbial water quality. Aquatabs usage fell by 50% after behavioral promotion visits concluded, suggesting intensive promotion is necessary for sustained uptake. The study findings suggest high potential for an automated decentralized water treatment system to increase consistent access to clean water in low-income urban communities.

## Introduction

Centralized disinfection of drinking water in urban settings has been shown to significantly reduce infectious disease; there is evidence that the introduction of filtration and chlorination in cities explains half of the reduction in child mortality during the 1900’s in the United States [1]. It has proven difficult, however, to provide clean drinking water employing centralized treatment systems in low-income urban areas, particularly those with intermittent water supply [2–4]. For these settings, centralized treatment is often ineffective because low water pressure and leaking pipes allow sewage to be drawn into the water distribution system [3,4]. In Asia only one-half of piped systems are estimated to supply water continuously [5]. Thus, while global access to improved water sources (*e*.*g*. public standpipes) is high (89%)[6], a large proportion of these improved sources actually provide water contaminated with sewage or feces, affecting an estimated 1.2 billion people global [7].

In-home disinfection technologies such as biosand filters, ceramic pot filters, chlorine kits, and UV light systems have been developed and promoted in an attempt to address the challenge of providing high quality water at the point of use (POU[8]. Despite the demonstrated efficacy of POU technologies in field trials with active promotion and in emergency settings[9,10], household demand for these products remains low, sustained use is rarely achieved, and scale-up has proven difficult [11–14]. A main disadvantage to POU technologies is they require new habit formation by the user, often requiring a substantial time burden on a daily basis [11,15]. In low- and medium-income countries, only 11% of households report practicing household water treatment with a POU product [16]. In Dhaka, Bangladesh, studies have shown that even short-term usage (3 months) of POU products (*e*.*g*. filters, chlorine tablets) provided free of charge is less than 30% [15]. Moreover, POU uptake is strongly associated with wealth, meaning that low-income households most at risk of water- and sanitation-related illness are those least likely to use POU products [16].

To date there has been limited effort to explore community-level options for water disinfection that would avert the need for household-level behavior change. Urban municipalities wishing to invest in water treatment have few options appropriate for low-income settings. For example, chlorination is widely considered the most cost-effective option for disinfecting water, yet most automated chlorine disinfection products on the market are not appropriate for water points that have intermittent supply, low flow rates (<5 L/s), and unreliable electricity supply. Water treatment technologies that rely on continuous electricity and continuous water supply are not well suited to low-income communities.

This paper describes a pilot study to evaluate a novel low-cost technology designed to dispense sodium hypochlorite automatically (passively) at shared handpumps in low-income areas of Dhaka, Bangladesh. The device fits onto manual handpumps connected to the municipal supply, chlorinating water at the end of the distribution system. The concept behind the passive chlorinator technology is to shift the focus of safe water innovations to the point of collection, overcoming the high cost and vulnerability of centralized treatment to recontamination during distribution, as well as removing the burden of daily behavior change required of households to treat their water with point-of-use treatment products. We evaluate this community-level passive chlorine dosing system in comparison to the *status quo* water supply provided by the municipality, and to a POU chlorination intervention consisting of the provision of water chlorination tablets (Aquatab), safe water storage containers, and behavior change promotion. Our main outcomes of interest include levels of free chlorine residual and fecal indicator bacteria in source and household stored drinking water, as well as user satisfaction.

## Materials & Methods

### Study site and sample frame

In Dhaka, a compound is a low-income housing community typically consisting of five to fifty households who share a common landlord, cooking area, toilet(s), and water collection point(s) within the compound boundaries. Water points are typically situated within compounds and owned by landlords to service the compound residents. Field staff recruited compounds into the study in August and September 2012 in low-income neighborhoods of the Mirpur sub-district in Dhaka. All compounds met the following eligibility criteria: (1) their primary drinking water source was one or two handpumps located within the compound and connected to a municipal water line operated by Dhaka Water Supply and Sewage Authority (DWASA); (2) they included at least three households with one or more children under the age of five years; (3) they included no more than thirty households in total; (4) they had not previously participated in a research study involving water treatment or hygiene; (5) their handpumps were not visible from another compound’s handpump; and (6) their handpumps were at least 10 meters apart from handpumps in other compounds.

### Ethics

Field staff obtained written informed consent from a representative of each household, and from the compound landlord, prior to enrollment in the study. The Ethical Review Committee at the International Center for Diarrheal Diseases Research, Bangladesh (icddr,b) (PR-09048) and the Institutional Review Board at Stanford University (IRB-22052) approved the study protocol.

### Baseline

Field staff conducted interviews with mothers (or the female primary caregiver) among all households with children under five in the enrolled compounds to gather basic household information, and to assess perceptions of drinking water quality and water supply services overall. We selected households with children under five to participate in data collection because this age group is the most vulnerable to death from waterborne illness, and we wanted to evaluate the interventions among households with children in this vulnerable age group. To test for fecal contamination and determine baseline levels of chlorine concentrations, staff collected samples from each handpump and from household stored drinking water.

### Intervention delivery

Compounds were sorted by size (number of households) and grouped into 5 strata consisting of 4 compounds each, then a randomization ratio of 2:1:1 was used within each stratum to determine assignment to one of three treatment groups: (1) passive chlorinator installed at handpump, (2) chlorine tablets and safe water storage containers, and (3) control (no intervention). Twice the number of compounds was assigned to the passive chlorinator group, since one of the main study objectives was to evaluate effectiveness and acceptability of the device for the first time. Twenty eligible compounds were enrolled into the study; ten compounds were assigned to receive a passive chlorinator, five compounds were assigned to chlorine tablets and safe storage containers, and five were assigned to the control group.

The passive (automatic) chlorinator was conceptualized and prototyped in the lab at Stanford University, then field-tested and modified in Dhaka, Bangladesh. A scoping survey of 127 water points among 45 slums in low-income communities in Dhaka conducted by our research team revealed that almost all (93%) of slum residents were accessing water points connected to the municipal system, with the most common method of extraction being manual handpumps (61%). Water points were also found to be conveniently located (median reported walk time = 0 minutes), and 87% of water points were situated within compounds and owned by landlords. The passive chlorinator was thus designed to be compatible with manual handpumps for the purposes of this study, however the technical concept could be simply adapted to also be compatible with taps connected to the piped distribution system.

**Fig 1:**
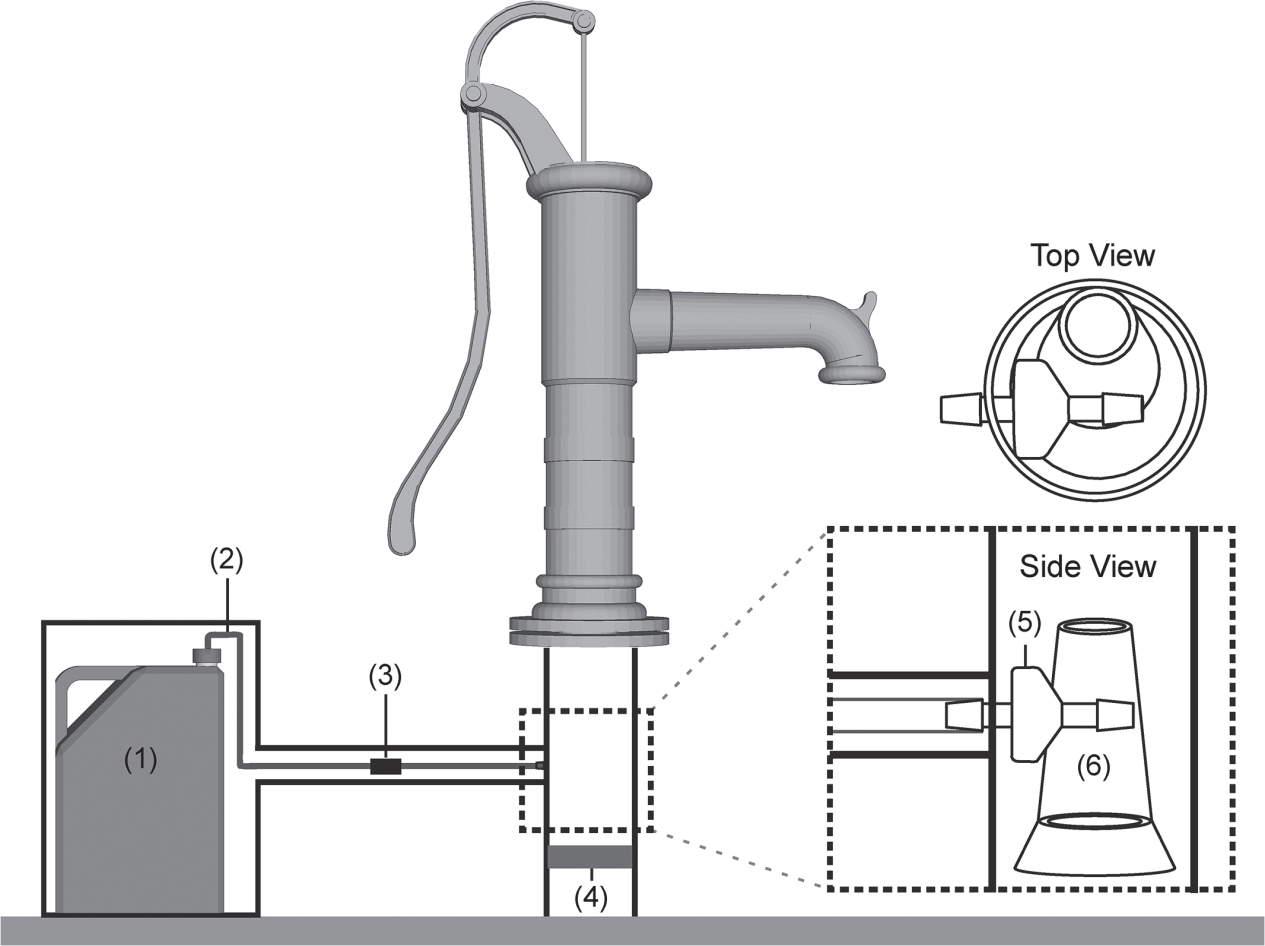
Schematic of passive chlorinator. The device consists of (1) 10-liter chlorine reservoir (plastic jerrycan); (2) plastic flexible tubing (1/8 inch inner diameter) connecting the reservoir to the injection point; (3) a regulator to control the chlorine dose; (4) a brass non-return valve placed within the pipe, below the handpump and underneath the injection point; (5) two non-return valves at each end of the tubing (one shown located at the injection point and one hidden located just inside the chlorine reservoir outlet); and 6) a plastic funnel inserted into the pipe below the handpump to direct the flow past the injection point. Side view and top view show pipe cross-section with chlorine injection valve and funnel positioning.

The device relies on the suction created by each handpump stroke to pull chlorine through an injection point in the pipe underneath of the handpump (see schematic diagram in Fig. 1). The dosing system has no moving parts and does not require electricity. The device consists of 1) 10-liter chlorine reservoir (plastic jerrycan); 2) plastic flexible tubing (1/8 inch inner diameter) connecting the reservoir to the injection point; 3) a regulator to control the chlorine dose, an off-the-shelf product manufactured for IV drips in hospitals (TrueCare Biomedix, Miami, FL); 4) a brass non-return valve placed within the pipe, below the handpump and underneath the injection point; 5) two non-return valves at each end of the tubing (one located at the injection point and one located at the chlorine reservoir outlet); and 6) a plastic funnel inserted into the pipe below the handpump to direct the flow past the injection point. The total cost of materials to construct one device was US$23. A security box with padlock (US$14) was built locally to keep the device and chlorine reservoir protected from UV exposure and secure from theft or tampering. All parts were sourced from Dhaka except for the regulator, tubing, and non-return valves. The device was branded with the Bengali phrase “Nirapad Pani,” translated as “safe water.” Prior to installations, extensive field-testing verified that the device delivered a constant dose over a range of handpump typical flow rates (0.1–0.4 L/s). The chlorine dosing devices were installed at each handpump with the assistance of local handpump mechanics (labor cost $2.50 per installation) and adjusted to deliver a target dose of 1 mg/L free chlorine residual. This dosing level was determined to be acceptable to users during pilot installations.

Chlorine refills (0.3% sodium hypochlorite) were delivered to each device by study staff at least once per week. As part of planned maintenance, minor adjustments to the regulator setting on each device were made to optimize dosing throughout the study during chlorine refill visits. At month 4, it was noted by field staff that air might be entering the system through the dosing regulator. To improve dosing performance, a technical adjustment to all passive chlorinators was performed at this time, which involved submerging the IV regulator in the chlorine reservoir in order to minimize air entering the system.

The in-home chlorine intervention consisted of regular supply of Aquatabs (Medentech, Wexford, Ireland), water disinfection tablets each containing 33mg of Sodium Dichloroisocyanurate (NaDCC). Each Aquatab tablet delivered a dose of 2 mg/L free chlorine residual per 10 liters of water. Based on findings from a previous study promoting Aquatabs with successful uptake rates in rural Bangladesh [17], each household also received a 10-liter safe water storage container, plastic stool to support the container, cleaning brush, and detergent. Tablets were replenished every two weeks by study staff.

A few days prior to hardware delivery, an intervention promoter held compound-wide meetings with all residents in study compounds to introduce them to chlorinated water and the potential health benefits, as well as give instructions on how to use the products. After hardware delivery, the promoter subsequently visited those households with at least one child under five in both intervention groups at regular intervals, for a total of five visits per household over a 4-month period.

### Follow up

Two of the 10 compounds assigned to receive a passive chlorinator withdrew from the study before installation was complete. One was excluded because they insisted on receiving the safe storage containers delivered as part of the Aquatab intervention (but not included as part of the passive chlorinator intervention). The second withdrew after the device security box was installed but prior to commencing chlorine dosing; the compound landlord cited taste-related objections as the main reason for withdrawing, even though the handpump water had not yet been chlorinated. These compounds are not included in the primary analysis because the landlords did not consent to the intervention and withdrew from data collection.

**Fig 2:**
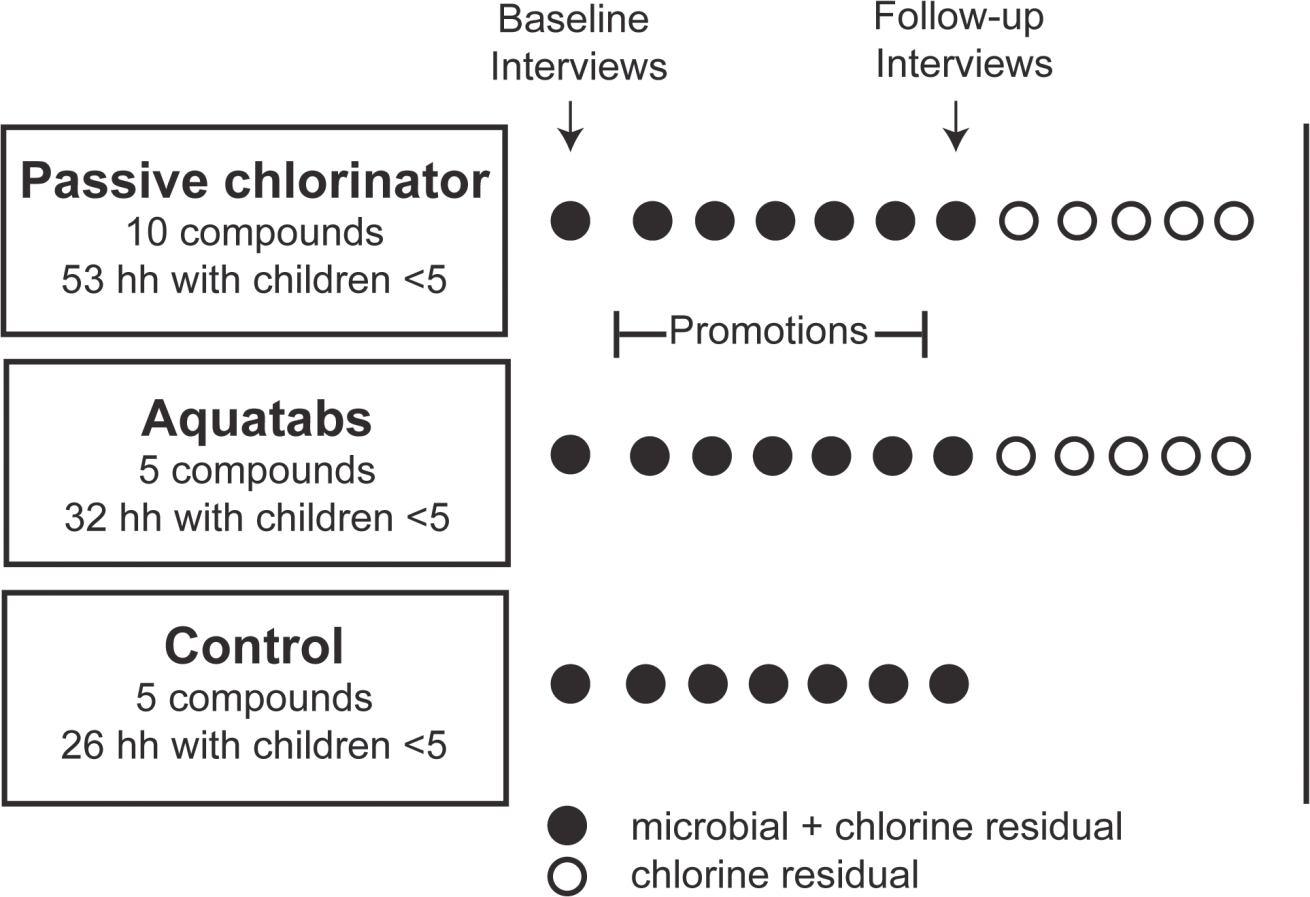
Data collection schedule for households (hh) with children under five years of age in study compounds. Solid black circles indicate water sampling for microbial and chlorine residual analysis, open circles indicate water sampling for chlorine residual analysis only. The duration of the follow-up data collection period (post-baseline and hardware delivery) was 10 months. Interviews were conducted at baseline and at five months of follow-up.

Primary data collection was focused on households with children under five (although all households residing in intervention study compounds received the chlorination hardware). After the baseline visit and hardware delivery, field staff (different staff from those who participated in promotion activities) conducted 11 follow-up visits with each intervention household that had a child under five years old. The data collection visits were conducted at regular intervals during a 10-month follow-up period to track uptake of the interventions. Follow-up visits were also conducted at the same frequency in the control group, except data collection ceased in the control group after month 5 (the 6^th^ household follow-up visit) due to minimal observed changes in water quality. At each follow-up visit, handpump and stored drinking water were tested for levels of total and free chlorine. For the first 5 months of follow-up, samples of stored water were collected from each household as well as from the compound handpump to test for *E*. *coli* and total coliform. At month 5, a household interview was conducted with female respondents at households that had at least one child under five to assess satisfaction with the intervention products, including taste and smell perceptions. Fig. 2 shows the data collection schedule for households with children under five.

During follow-up months 5 through 10, chlorine residual was also measured in stored drinking water among intervention households that did not have children under five and who had not received any household-level behavior change promotion. Data from these households without children under five were not included in the primary analysis, but were analyzed separately to understand uptake among households not receiving household-level promotion.

### Water quality analysis

Field staff collected all water samples in sterile Whirlpak bags containing sodium thiosulfate. Handpumps were flushed by pumping prior to water sample collection from the outflow. Field staff asked respondents to extract household stored drinking water in the usual manner and pour into the sterile bag. Free and total chlorine of water was measured in the field using a digital colorimeter (LaMotte Model 1200, LaMotte Company, Chestertown, MD) [18]. All water samples collected were placed in an ice cooler after collection, then filtered within six hours of collection at the environmental microbiology laboratory at icddr,b. *E*. *coli* (an indicator of fecal contamination) and total coliform (an indicator of treatment efficacy) concentrations were enumerated using membrane filtration following US EPA Standard Method 1604 [19]; 100mL of each sample was filtered through a 0.45 micrometer filter, then placed on MI media and incubated at 35 degrees Celsius for 24 hours. Plates with 500 or more colonies were deemed too numerous to count, following previously published protocols for water quality testing in low-income settings [20–22]. One duplicate sample was run for every ten samples collected; one lab blank was run per day.

### Data analysis

Microbial water quality samples under the detection limit were assigned the value of 0.5 CFU/100mL and samples above the detection limit were assigned the value of 500 CFU/100mL. Detectable total chlorine was selected as an indicator of whether or not water had received chlorination, and serves as the primary indicator of adoption. A detectable level of chlorine residual was considered to be greater than or equal to 0.1 mg/L based on the detection limit of the colorimeters. Poisson regression was used to model binary water quality parameters in stored drinking water by treatment group, including presence/absence of detectable total chlorine residual; presence/absence of detectable free chlorine residual; presence/absence of a chlorine residual above 0.2 mg/L (the minimum protective residual according to the World Health Organization); presence/absence of *E*. *coli* contamination; and presence/absence of total coliform contamination. Poisson regression was chosen to allow for prevalence ratios to be calculated, which are easier to interpret than odds ratios. The association between chlorine residual levels and storage time was modeled with linear regression; associations between the presence of chlorine residual and presence of bacterial contamination were modeled with logistic regression. Finally, to assess differences in household satisfaction, usability, and user perceptions, logistic regression was employed. All regression models include robust standard errors (by the Huber-White sandwich estimator) to account for clustering at the compound level; water quality models also control for study duration by including study month. Analysis was done in STATA v12. We considered P-values below 0.05 statistically significant (unlikely to be due to chance).

## Results

### Baseline

A total of 354 households resided in the twenty study compounds (177 households were assigned to the passive chlorinator group, 90 to Aquatabs, and 87 to control); 32% of households had children under five. Baseline interviews were conducted with mothers (mean age 27 years) from the 113 households that had at least one child under five (N = 55 assigned to passive chlorinator, N = 32 assigned to Aquatabs, and N = 26 assigned to control). Only 26% of respondents reported completing primary school. Compounds had an average of 18 households, with mean household membership of 4.6 people. Median household income was 10,000 taka ($125 USD) per month.

Each handpump supplying drinking water was used by an average of 19 households. One-third of households reported using a secondary water source for domestic water needs other than drinking water; 95% of these secondary sources were also handpumps connected to the municipal water line. Almost all households (95%) reported that payment for access to the handpump was included with their monthly rent. The majority (79%) of respondents reported satisfaction with the handpump water taste. When asked how safe it was to drink water directly from the handpump, 43% of respondents thought it was “very safe,” 27% reported “somewhat safe,” and 28% thought the water was “unsafe.” Only 10% of respondents thought that drinking untreated water from the handpump makes household members sick, and 89% of households reported never treating their drinking water. Very few households had experience with chlorine products for disinfecting drinking water; fewer than 3% reported having consumed chlorinated water previously. Only 4% had heard of using liquid chlorine to treat drinking water, and one-third had knowledge of chlorine tablets.

At baseline, 75% (N = 24) of handpumps tested positive for contamination with *E*. *coli* (median 3 CFU/100mL); total coliform contamination was universal with more than 500 CFU/100mL enumerated from one-fifth of handpumps (median 153 CFU/100mL). Stored drinking water tested positive for *E*. *coli* at 86% (N = 112) of households (median 7 CFU/100mL), and more than half of households had more than 500 CFU/100mL total coliform (median 500 CFU/100mL). There were no significant differences in water quality parameters between treatment groups at baseline (all p>0.05).

### Follow up—chlorine residual in source water

**Fig 3:**
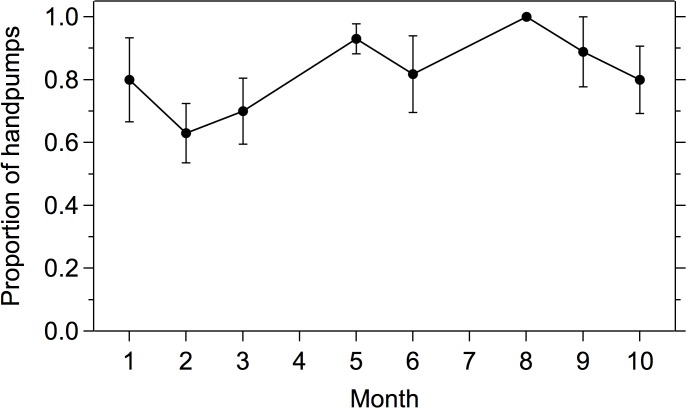
Proportion of samples from handpumps fitted with a passive chlorinator delivering water with detectable total chlorine residual (>0.1mg/L) over a 10-month follow-up study period. Error bars show 95% confidence interval.

Handpumps fitted with passive chlorinators were found to have detectable total chlorine residual in 80% (N = 129) of samples collected; in the Aquatabs and control groups samples collected directly from handpumps had total chlorine residual in 7% (N = 68) and 0% (N = 31). On average, the passive chlorinators delivered a dose of 0.75 mg/L (SD 0.61) total chlorine and 0.66 mg/L (SD 0.57) free chlorine to handpump water. Fig. 3 shows the proportion of passive chlorinators successfully delivering chlorinated water over time; improved dosing performance (mean levels of 0.96 mg/L total chlorine and 0.85 mg/L free chlorine) was observed after the technical adjustment at month 4.

### Follow up—chlorine residual in household stored water

Households with children under five receiving the passive chlorinator and Aquatabs interventions were significantly more likely to have total and free chlorine residual detected in their stored drinking water (P<0.001, Table 1) compared to the control group. Among all follow-up visits, 75% (N = 413) of household visits in the passive chlorinator group found detectable total chlorine residual (>0.1mg/L) in the stored drinking water, compared to 72% (N = 336) of household visits in the Aquatabs group, and to 6% of household visits in the control group (N = 119).

**Table 1:** Modeling results (Poisson regression) comparing quality of stored drinking water among households with children under-five between treatment groups.

		Passive chlorinator *vs*. control		Aquatabs *vs*. control		Passive chlorinator *vs*. Aquatabs		Passive chlorinator *vs*. Aquatabs (months 5–10 only)		
	N	PR (95% CI)	p-value	PR (95% CI)	p-value	PR (95% CI)	p-value	N	PR (95% CI)	p-value
Total chlorine	868	13.5 (6.8–27.1)	<0.001	13.0 (6.4–26.1)	<0.001	1.0 (0.9–1.3)	0.634	401	1.5 (1.1–2.1)	0.004
Free chlorine	868	17.3 (8.5–35.5)	<0.001	18.7 (9.1–38.0)	<0.001	0.9 (0.8–1.1)	0.480	401	1.3 (0.99–1.8)	0.060
Free chlorine >0.2mg/L	868	35.8 (12.7–100.8)	<0.001	46.7 (16.6–131.0)	<0.001	0.8 (0.6–0.9)	0.011	401	1.1 (0.8–1.6)	0.397
*E*. *coli*	538	0.33 (0.22–0.48)	<0.001	0.25 (0.17–0.38)	<0.001	1.3 (0.9–2.0)	0.219		N/A	
Total coliform	538	0.58 (0.52–0.65)	<0.001	0.44 (0.37–0.53)	<0.001	1.3 (1.1–1.6)	0.015		N/A	

Prevalence ratio (PR) and 95% Confidence Interval (CI) shown for each comparison between groups; all models include study duration and clustered standard errors at the compound level Households in Aquatab compounds reported storing their water a median of 8 hours, while households in passive chlorinator compounds reported a median of 4 hours. Longer storage time was significantly associated with decreased free chlorine residual levels among passive chlorinator compounds (P = 0.011), but was not associated with chlorine residuals among Aquatab compounds (P = 0.215).

There were no significant differences in the proportion of household stored water samples with detectable total chlorine and detectable free chlorine residual between Aquatab (total 75%, free 68%) and passive chlorinator compounds (total 72%, free 72%) (Table 1). However, the proportion of household stored water samples with free chlorine residual above 0.2 mg/L was significantly higher among Aquatab compounds (71%) compared to passive chlorinator compounds (55%) (Table 1). The mean level of total chlorine residual in stored drinking water was also significantly higher in Aquatab households (1.04 mg/L) than in passive chlorinator households (0.46mg/L) (P<0.001).

**Fig 4:**
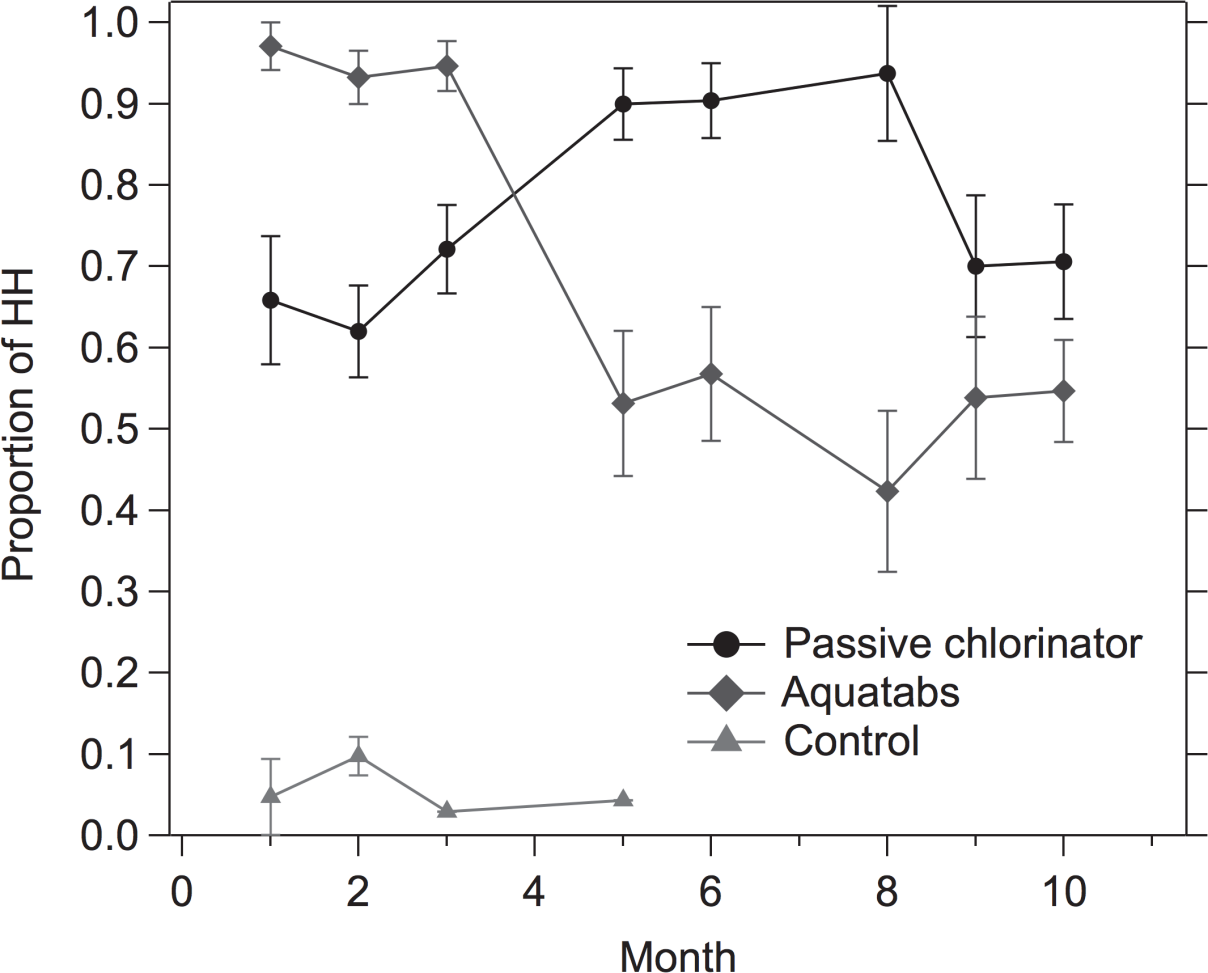
Proportion of households with detectable total chlorine residual in their stored drinking water (>0.1mg/L) by treatment group spanning a 10-month follow-up period. Behavior promotions ended after month 4. Control group was not sampled past month 5. Error bars show 95% confidence interval of mean proportions.

While biweekly behavioral promotions were ongoing, Aquatab usage was over 90% (as measured by presence of total chlorine residual) (Fig. 4). After regular promotional visits concluded, Aquatab usage dropped by 50% (after month 4 in Fig. 4). Following the technical adjustment to the passive chlorinators at month 4, the likelihood of detecting chlorine residual in the passive chlorinator group increased (Fig. 4). Time trends of the proportion of households with detectable chlorine residual correspond with dosing time trends of the passive chlorinator (Fig. 3, Fig. 4). In addition, households in the passive chlorinator compounds did not report collecting their stored drinking water from handpumps outside their compound significantly more often than households in Aquatab compounds (7% *versus* 5%, χ^2^ = 1.6, P = 0.20), indicating that very few households switched their primary drinking water source during the intervention period.

When analyzing the subset of data collected during the second half of the study after behavior promotion visits concluded but chlorine and Aquatab refills continued to be delivered for free (during months 5–10 of follow up), households in passive chlorinator compounds were 1.5 times more likely to have detectable total chlorine residual in their stored water than households in Aquatab compounds (95% CI 1.1–2.1, P = 0.004). Households in passive chlorinator compounds that did not have children under five (and that had not received any household-level promotional visits) were 1.9 times more likely (95% CI 1.2–3.0, P = 0.009) to have a detectable total chlorine residual in their stored drinking water (75%), compared to households without children under five in Aquatab study compounds (40%).

### Follow up—microbial water quality

**Fig 5:**
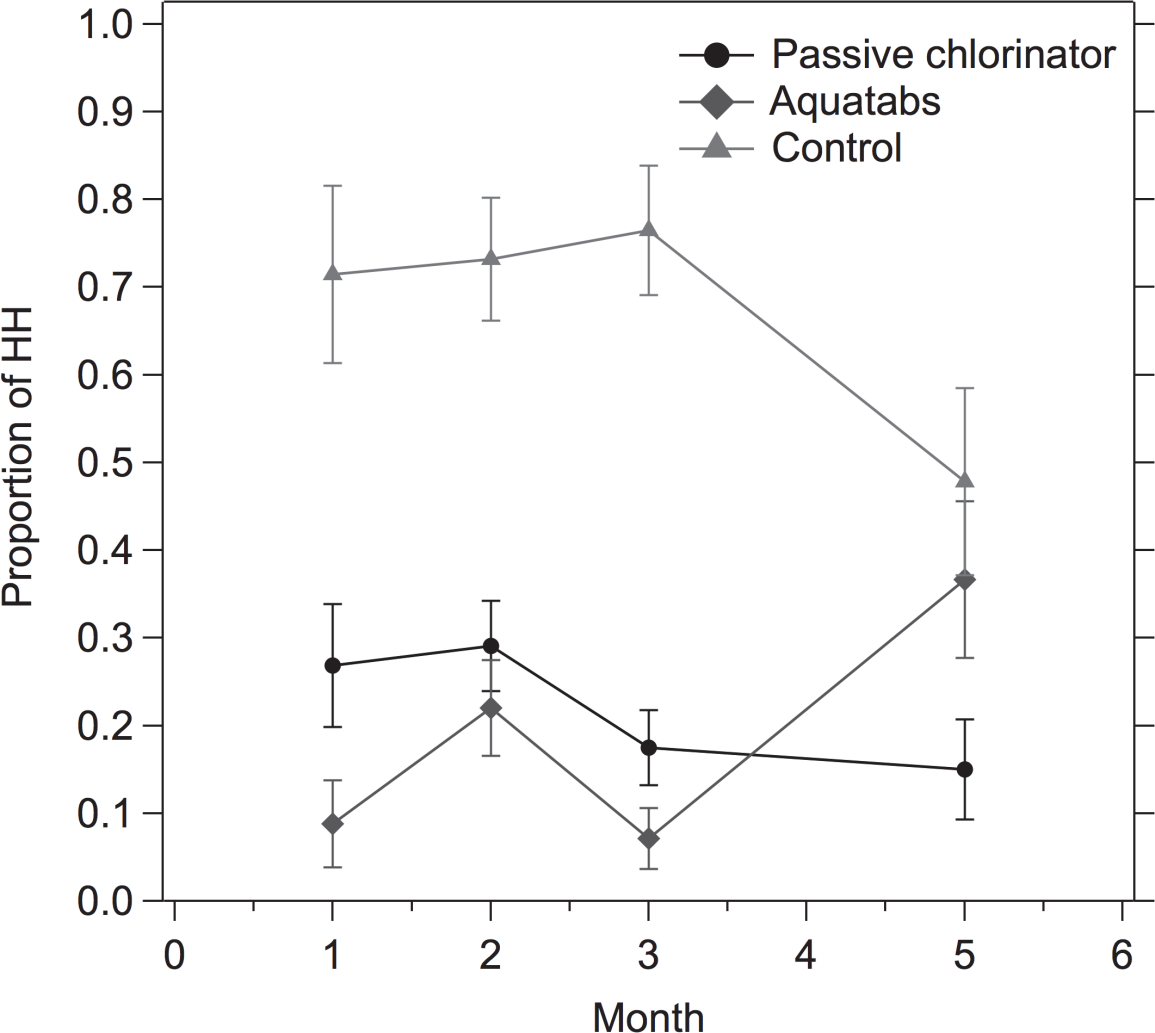
Proportion of households with detectable *E*. *coli* contamination in their stored drinking water by treatment group spanning a 5-month follow-up period. Error bars show 95% confidence interval of mean proportions.

Households in both the passive chlorinator and Aquatabs groups were significantly less likely to have *E*. *coli* or total coliform contamination in their stored drinking water compared to the control group (N = 538, p<0.001, Table 1, Fig. 5). Household stored drinking water in the passive chlorinator group was free from *E*. *coli* contamination 78% of the time and had an overall median of 7 colony forming units (CFU) *E*. *coli* per 100mL (N = 240). Household stored drinking water in compounds receiving Aquatabs was free from *E*. *coli* contamination 83% of the time and had a median of 6 CFU *E*. *coli*/100mL (N = 179). In control households stored drinking water samples had a median of 46 CFU *E*. *coli*/100mL and 31% were free from *E*. *coli* (N = 119). None (0%) of the stored water samples collected from control households, 66% from Aquatab households, and 42% from passive chlorinator households were free from total coliform contamination. There was no significant difference in *E*. *coli* contamination between the passive chlorinator and Aquatab groups (Table 1); however, household stored water in the Aquatabs group was significantly less likely to have total coliform contamination than household stored water in the passive chlorinator group (p = 0.015, Table 1). The presence of *E*. *coli* and total coliform contamination was significantly and negatively correlated to detectable total and free chlorine residual (p<0.001). However, 8% of water samples with detectable free chlorine residual had *E*. *coli* contamination and 40% of these had total coliform contamination.

### Follow up—household perceptions

A total of 93 households with children under-five were interviewed at month 5 of follow up; given the highly transient urban population there were less households with children under-five available for interview at month 5 than at baseline. When questioned, 70% of passive chlorinator users (N = 40) and 83% of Aquatab users (N = 30) were generally satisfied with their water treatment products (P = 0.23). When asked about ease of use, 50% of passive chlorinator users reported that the device was “very easy” to use, while 50% reported it was “easy” to use. In contrast, 3% of Aquatab users reported that the tabs were “very easy” to use, while 97% reported that they were “easy” to use (P<0.01). Satisfaction with taste was similar across both intervention groups; 75% were satisfied with the taste of passively chlorinated water, and 77% were satisfied with the taste of Aquatab treated water (P = 0.89). Satisfaction with smell was higher among households using Aquatabs (70% satisfied) compared to households using the passive chlorinator (45% satisfied) (P = 0.14). The majority of passive chlorinator users (83%) and Aquatab users (83%) reported that their household preferred to drink water treated with the chlorinator/Aquatabs compared to untreated water.

## Discussion

This study demonstrated that a novel passive chlorinator could perform as well as a POU water chlorination intervention in increasing access to chlorinated drinking water. Over a 10-month intervention period, the share of households with stored chlorinated drinking water remained above 60% in compounds with access to the passive chlorinator and above 40% in compounds receiving Aquatabs (Fig. 4). In addition, both the passive chlorinator and POU intervention significantly improved household stored microbial water quality, as compared to the control group (Table 1). However, after behavioral promotion visits concluded, over 50% of compliant households immediately stopped using chlorine in Aquatab compounds. Use of chlorinated water did not decrease in passive chlorinator compounds after promotions ceased. These results suggest regular intensive household-level promotion is necessary for sustained use of a household (point-of-use) water treatment intervention, but would not be required for scale-up of passive chlorination. There is potential for automated decentralized water treatment systems to increase consistent access to clean water in low-income urban communities at low-cost.

Comparing adoption rates in the passive chlorinator and Aquatab (POU) intervention compounds reveals advantages and disadvantages of each approach. Two compounds withdrew from the passive chlorinator group prior to intervention delivery; in these cases the landlord made the decision to withdraw, highlighting the authority of landlords in initial compound-level adoption of the passive chlorinator. Despite some moderate reported dissatisfaction with the smell of chlorinated water, households did not switch their primary drinking source to avoid water treated by the passive chlorinator. Among households that did not receive promotional visits (without children under five), those in the passive chlorinator compounds were two-fold more likely to have chlorinated stored drinking water in their home compared to those in compounds receiving Aquatabs without promotional visits. Adoption rates of chlorinated water were similar across all households in passive chlorinator compounds, indicating that adoption of the passive chlorinator is not dependent on intensive household-level promotion. More broadly, adoption of the passive chlorinator depends on successful performance of the technology and initial acceptance by landlords, while adoption of Aquatabs relies on successful sustained behavior change at the household-level.

Although the passive chlorinator underwent extensive field-testing prior to the launch of the study, its dosing performance was not as consistent as expected. It is theoretically possible that a more refined product would have substantively outperformed the household-level chlorination intervention in terms of increasing sustained use of chlorinated drinking water among households. The effectiveness of the passive chlorinator in this study could be considered a lower-bound for the potential of this novel decentralized and automated approach to improve water quality, contingent on further technical improvements to the device.

The passive chlorine dosing devices did not maintain a dose of 1.0mg/L free chlorine residual throughout the 10-month intervention period with 100% consistency. Several factors could explain the observed variation in dosing. First, submerging the regulator at month 4 improved dosing during months 5–10 (Fig. 3). Second, water availability was reduced in the study area due to uncharacteristically low water pressure in the municipal piped system during months 9–10 (confirmed by personal communication with a municipal pump operator), which may have altered the suction dynamics of the device (see months 9–10 in Figs. 3 & 4). Third, the IV regulators exhibited clogging intermittently throughout the study (which was addressed by opening and flushing the regulator during chlorine refill visits), potentially due to buildup of solid precipitate from the concentrated chlorine solution. Fourth, variation in chlorine demand of the source water could have contributed to variability in measured chlorine residual [23]. Finally, a municipal water distribution pipe was replaced near one of the compounds, which resulted in a dramatic increase in water pressure that turned the handpump into a free-flowing tap. Since the passive dosing device relies on the suction created by the handpump stroke, dosing at this compound was no longer possible during months 9–10. Based on the data collected during this study, the passive chlorinator would benefit from further development to improve dosing reliability.

Adoption of Aquatabs in this study exceeded usage reported by previous intervention studies, despite the noticeable drop in usage during the second half of the study. In comparison, Luoto *et al*.(2011) report an uptake rate of 10% in a low-income community in urban Dhaka (as measured by a positive chlorine residual test) [15], while Boisson *et al*. (2013) report an uptake rate of 32% in a rural community in India [24]. The high quality promotional materials and up market imported safe water storage container delivered with the Aquatabs intervention may have contributed to the high adoption rate; these intervention materials had the benefit of extensive previous field testing by Ercumen et al. in rural Bangladesh [17]. The safe storage container may have prevented recontamination, contributing to the observed greater reduction in total coliform among Aquatab compounds. Notably, the biweekly promotional visits had a substantial influence on Aquatab usage; when promotional visits stopped after survey round 5, uptake of Aquatabs fell by half, from >90% to between 40–60% for the remainder of the study (Fig. 4). These findings emphasize the difficulty in achieving consistent and sustained use of POU products without ongoing intensive behavioral promotion.

User perceptions differed between the passive chlorinator and Aquatab groups. Although satisfaction with taste in both groups were similar, households in the passive chlorinator compounds were less likely to be satisfied with the smell of the treated water than those receiving the Aquatab intervention. Considering the passive chlorinator removes the user from the dosing process, the reason for this smell perception difference is unclear. However, it may result from the sodium hypochlorite (diluted household bleach) used in the passive chlorinator compared with the sodium dichloroisocyanurate in Aquatabs. Households in passive chlorinator compounds were much more likely to perceive usage as “very easy,” suggesting that the passive chlorinator achieved the goal of requiring minimal behavior change from the user.

Several study limitations should be acknowledged. First, the passive chlorinator was designed to dose liquid sodium hypochlorite at 1 mg/L free chlorine residual, while Aquatabs provide a dose of 2 mg/L using sodium dichloroisocyanurate. These differences in active ingredient and target doses likely influenced the effectiveness and adoption rates of the two interventions, and should be considered when interpreting the results from this study. A higher target dose by the passive chlorinator could have resulted in a higher proportion of households accessing water with a free chlorine residual above 0.2mg/L. Second, attrition from the study was differential across treatment groups since two of the compounds assigned to receive a passive chlorinator withdrew from data collection prior to delivery of the intervention (and no compounds withdrew from the Aquatab or control groups). Continued promotion and data collection at these two compounds was not feasible, but likely would have resulted in reduced preference and lower overall uptake rates of the passive chlorinator. However, the higher initial refusal rate of the passive chlorinator compared with household-level interventions could be economical for scale-up if installations are reserved for those compounds that will be more receptive to the device. Third, Aquatabs are not currently available in Bangladesh. Other locally available POU chlorination products could result in lower adoption rates. Finally, the household interview sample size was small, which may have impacted the utility of statistical tests to determine if the differences between groups in perceptions of taste, smell, and satisfaction were larger than would be expected by chance.

Developing a business model to ensure cost recovery for the passive chlorinator was outside the scope of this study, but would be critical for successful scale-up. Our best estimates indicate that the cost of operating the passive chlorinator, including regular delivery of chlorine refills, would be $0.10 USD per household per month, contingent on local sourcing of high quality chlorine and assuming 15 households served per installed device. A potential fee recovery system could target landlords owning shared water points to recover user fees through household monthly rents, as monthly service fees per household would be less than 1% of typical monthly rents.

Rising urbanization and population growth is changing the landscape for water treatment technologies. The number of urban low-incomes households relying on networked intermittent water supply systems is increasing [4,6]. This shift is opening up new opportunities for technology development at an intermediate scale, between city-wide (centralized) and household-level treatment. Community-level (decentralized) treatment systems have the potential to be more affordable than household-level treatment by serving more households. Development of small-scale automated water treatment systems could also lead to increased coverage and sustained use, since they do not require changes in daily behavior by every household, a main barrier to scaling up POU approaches [11,13]. The design of community-level automated systems that do not rely on household-level behavior change and that are appropriate for disinfecting water in settings without reliable electricity should be considered a new technology development challenge for increasing access to safe water.

## Supporting Information

S1 CONSORT Checklist CONSORT checklist for reporting randomized trial. (DOC)

S1 Dataset (DTA)

S1 Flow Diagram CONSORT participant flow diagram. (DOC)
