# Supplementary material for: Differences in Field Effectiveness and Adoption between a Novel Automated Chlorination System and Household Manual Chlorination of Drinking Water in Dhaka, Bangladesh: A Randomized Controlled Trial
Source: PLoS One. 2015 Mar 3;10(3):e0118397. doi: 10.1371/journal.pone.0118397 (PMC4348460; doi:10.1371/journal.pone.0118397)
Supplement: S1 Flow Diagram — (DOC) [file pone.0118397.s003.doc]

**CONSORT Flow Diagram**

Assessed for eligibility

(n= 33 compounds)

Excluded (n = 13 compounds)

♦  Not meeting inclusion criteria (n= 13)

♦  Declined to participate (n= 0)

Analysed (n= 5 compounds)

Lost to follow-up (n= 0 compounds)

Allocated to control (n= 5 compounds)

Lost to follow-up (n= 0 compounds)

Allocated to passive chlorinator intervention (n=10 compounds)

♦ Received passive chlorinator intervention (n= 8 compounds)

♦ Withdrew (n= 2 compounds)

Analysed (n= 8 compounds)

Randomized (n= 20 compounds)

Allocated to Aquatabs intervention (n= 5 compounds)

Lost to follow-up (n= 0 compounds)

Analysed (n= 5 compounds)
